# The Italian health data system is broken

**DOI:** 10.1016/j.lanepe.2024.101206

**Published:** 2025-01-03

**Authors:** The Lancet Regional Health – Europe

The population of Italy is projected to decrease by approximately 8% by 2050, falling from 59 million in 2022 to 54.4 million, due to increased ageing and a declining birth rate. By 2050, more than 35% of Italians will be older than 65 years, while children younger than 14 years will represent only 11.7% of the population. Without reforms, this demographic shift will strain health-care and social systems.

A major weakness of the health-care system in Italy is the fragmented health data infrastructure: there is no unified, centralised system for documenting and sharing electronic health records (EHRs), hospital data, and general practitioner records.

The root cause is extensive regional autonomy, with 20 regions operating independently and implementing differing policies and technologies, creating regulatory fragmentation and inefficiencies. Poor interoperability between regions and hospitals, in addition to the lack of automatic data upload systems in private clinics, undermines the effectiveness of the Fascicolo Sanitario Elettronico—Italy's national EHR system designed to track patients' health histories—rendering it largely ineffective due to these structural flaws.

Compounding this is the absence of a national policy to allocate resources equitably to all regions or establish standardised protocols for data collection and transfer. Many hospitals and facilities continue to rely on outdated, incompatible systems, making the transfer of patient records and diagnostic images manual and labour-intensive, even within the same region or city. The absence of standardisation prevents the creation of national registries, hampering effective care, and crisis management.

The consequences of this fragmented system are profound. During the COVID-19 pandemic, it delayed the identification of links between comorbidities and infection severity, exacerbating regional disparities in health-care capacity and outcomes. A better-integrated system could have enabled broader analyses, generalisable insights, and supported a more effective, coordinated national response.

Such a fragmented system not only fails the Italian population but also imposes a considerable economic burden on the country. Patients from southern regions, which are typically more resource limited, travel to better-equipped northern hospitals for treatment. However, due to the lack of interoperable systems, hospitals in the north often cannot access patient records, resulting in repeated diagnostic tests and delayed care. This duplication inflates costs—interregional health-care mobility alone accounts for around €3.3 billion annually—and undermines patient outcomes.

The fragmented health data system in Italy also presents considerable challenges for research. Without a central platform, researchers must apply to the ethics and privacy committees of individual institutions, which can deny requests without substantive scientific justification. Since 2009, the percentage of authorised studies out of the total has fallen to 15%, marking a significant decline. Furthermore, data collection is often manual and of poor quality, making multi-centre, high-quality studies nearly impossible to conduct, severely hindering the generation of impactful, generalisable findings.

In 2022, Italy spent €1.8 billion on digital health care, a 7% increase from the previous year. However, it remains unclear whether these funds have been fully utilised and how they were spent, particularly in relation to EHRs and the integration of regional and national health systems, since only 42% of clinics reported having an active electronic data capturing system in all departments.

Public distrust in the government exacerbates the issue, with over 90,000 Italians refusing to share their health data due to privacy concerns—a sentiment amplified during the COVID-19 pandemic. While Europe has embraced so-called legitimate interest legal basis, allowing health data to be used for research and innovation without relying solely on individual consent, Italy's restrictive legislation and regional fragmentation hinder these efforts, failing to balance privacy rights with the public interest to improve health care.

A newly proposed reform threatens to worsen the situation even further. The autonomia differenziata law, if passed, will further decentralise health-care governance, deepening the fragmentation and disparities between regions instead of fostering harmonised data collection and sharing.

Legislative harmonisation at the national level is essential to establish a unified health data network in Italy. This approach will support data interoperability, telemedicine, and the digitalisation of the Servizio Sanitario Nazionale (Italy's national health service), while leveraging European initiatives such as the Data Governance Act, which promotes secure and ethical data sharing, the European Health Data Space, which aims to enable cross-border health care and foster research, and the AI Act, which seeks to regulate trustworthy and transparent artificial intelligence in health care.

Failure to act will deepen inequalities, delay treatments, and hinder progress, whereas prioritising systemic reform offers Italy the opportunity to meet health-care demands and deliver fair, efficient care.

